# On the Location of Fog Nodes in Fog-Cloud Infrastructures

**DOI:** 10.3390/s19112445

**Published:** 2019-05-29

**Authors:** Rodrigo A. C. da Silva, Nelson L. S. da Fonseca

**Affiliations:** Instituto de Computação (IC), Universidade Estadual de Campinas (UNICAMP), Av. Albert Einstein 1251, 13083-852 Campinas, SP, Brazil

**Keywords:** fog computing, cloud computing, facility location, mixed-integer linear programming

## Abstract

In the fog computing paradigm, fog nodes are placed on the network edge to meet end-user demands with low latency, providing the possibility of new applications. Although the role of the cloud remains unchanged, a new network infrastructure for fog nodes must be created. The design of such an infrastructure must consider user mobility, which causes variations in workload demand over time in different regions. Properly deciding on the location of fog nodes is important to reduce the costs associated with their deployment and maintenance. To meet these demands, this paper discusses the problem of locating fog nodes and proposes a solution which considers time-varying demands, with two classes of workload in terms of latency. The solution was modeled as a mixed-integer linear programming formulation with multiple criteria. An evaluation with real data showed that an improvement in end-user service can be obtained in conjunction with the minimization of the costs by deploying fewer servers in the infrastructure. Furthermore, results show that costs can be further reduced if a limited blocking of requests is tolerated.

## 1. Introduction

The world has witnessed a massive growth in the number of devices connected to the Internet. The number of devices has already exceeded the world population [1], and it is expected to be from two to three orders of magnitude greater in the near future [2]. The Internet of Things (IoT) includes both user-dependent devices, such as smartphones and tablets, and user-independent devices, such as sensors and actuators. User-dependent devices can be mobile and can be connected to different networks at different times. Moreover, some services and applications used on these devices are latency sensitive, such as augmented reality applications.

Data generated by IoT devices have commonly been processed in cloud data centers [3], which provide computing and storage capabilities for resource-limited IoT devices. However, data centers are typically far from end users, which can lead to considerable delay in the processing of the IoT data and make certain applications unfeasible. One proposal to make such applications feasible is fog computing.

Fog computing was designed to support delay-sensitive applications as well as mobility by providing computing, networking, and storage capabilities at the edge of the network [4]. Fog computing fills the gap in service provisioning for latency-sensitive applications not considered by cloud computing. The fog is close to end users and processing on its devices allows the reduction of delays to only a few milliseconds. Moreover, fog is a distributed architecture, not centralized as is cloud computing. Fog computing was designed to complement the cloud, but not to replace it.

A fog-cloud infrastructure is useful in the execution of mobile applications consisting of tasks with different latency requirements [5]. One example is the augmented reality application in [6]. This application is divided into four tasks, and two of them should be processed in the fog due to their strict latency requirements. The fog enables novel applications, and it can also enhance the performance of typical cloud applications. In this example, the other two tasks with flexible latency requirements can also be processed in the fog rather than the cloud, thus reducing the total delay.

Fog nodes are the basic units for fog computing, and can be a network device which uses processing capabilities, dedicated servers, or computational servers to coordinate underlying devices [7]. A fog is usually composed of several levels of fog nodes, and the processing of a given application may be more adequate for a specific layer as a consequence of its requirements, such as latency, security, mobility, and scalability [5].

The number of levels in a hierarchical fog and the position of the nodes depend on the architecture involved. In the architecture described in [8], fog nodes are created near base stations in 5G networks, while, in the architecture described in [9], end users can provide residential fog devices and receive incentives to share the nodes. Previous papers [4,7,8,9] have discussed the role of fog nodes in the architecture and their connection to other network elements, but have not discussed the impact of the creation of fog nodes on different physical locations. The location of the fog problem consists in deciding where fog nodes should be placed given a set of potential locations and the devices available for deployment. The solution to the problem is crucial for fog providers. Indeed, the location decision affects both users and the provider. Wrong decisions can jeopardize user access: if the delay in accessing the fog impacts the application, user expectations will not be fulfilled. Moreover, the deployment of servers influences the costs of fog providers, reckless decisions can guarantee user satisfaction, but at a high deployment cost.

This paper proposes a solution for the fog node location problem. The problem is formulated as a mixed-integer linear programming (MILP) model which considers various inherent aspects of a fog-cloud system. To evaluate different classes of service, the model considers two types of demands: strict (which can only be processed in a fog node) and flexible (which can be hosted either in the fog or in the cloud). By considering these two types of workload, the solution attempts to serve requests which are dependent on the fog while improving the latency experienced by flexible applications. The solution was designed as a multicriteria optimization problem, focusing on the service of all demands at a reduced cost. Moreover, the demand of workload to be processed varies with time. A multi-level programming approach was employed to select a solution from the Pareto front, ordering the multiple objectives in a hierarchical manner. By evaluating variable demands, the solution captures the mobility of users.

Real data representing workload variation in geographical cells in a metropolitan area inhabited by mobile users were employed as the input to the problem formulation. Solutions were obtained using the hierarchical order of the objectives and variations allowing degradation in the objective functions were evaluated; this showed that reducing the quality of service in the service provisioning at a certain extent can lead to big savings in infrastructure costs.

This paper is organized as follows. Section 2 reviews related work on fog computing and facility location. Section 3 introduces the system model as well as the location problem. Section 4 presents the proposed solution and its mathematical formulation. Section 5 presents an evaluation of the solution and discusses related issues. Finally, Section 6 concludes the paper.

## 2. Related work

This section first reviews work on fog computing [4,8,9,10,11,12], and then presents papers dealing with the location problem [13,14,15,16,17,18,19,20]. Proposals for fog computing architectures are discussed in [4,8,9]. The main effort in this direction has been the OpenFog Consortium, which is in charge of defining an open and interoperable architecture for fog computing that organizes fog nodes in hierarchical layers [4]. The TelcoFog architecture [8] also considers layers, but it recommends that fog nodes powered with computational and storage resources should always be deployed next to cellular base stations. Another proposal [9] is a user-participatory architecture in which fog nodes are installed and owned by end users and leased to the provider to make the infrastructure scalable. These proposals [4,8,9] discuss the architectures of fog, but they do not specify the physical location of the fog nodes, even though this would impact on the service provided.

Another major issue in fog design is the definition of resource allocation mechanisms to manage fog resources [10,11,12]. A solution to support the QoS requirements of applications was proposed by Souza et al. [10] with a mechanism considering one cloud and two fog layers to reduce latency. Other proposals [11,12] decide on which layer (fog or cloud) a task from an application should be instantiated. The mechanism in [11] considers the history of previous arrivals to avoid overusage of fog nodes, thus achieving a good use of fog resources. The mechanism in [12] favors the creation of tasks in distant layers as long as the required latency is supported, but allows tasks to be rearranged in order to reduce the number of active nodes. Such mechanisms depend on the creation of fog nodes, but their location must be determined in advance.

Despite the proposals for fog architectures [4,8,9] and the allocation mechanisms for fog computing [10,11,12], the literature lacks solutions for deciding on the location of fog facilities. Some papers have addressed this problem for cloud data centers [13,14,15] and cloudlets [16,17]. Larumbe and Sansò presented solutions [13,14] to select the location of a data center in a backbone network. In [13,14], a MILP formulation and a scalable tabu search algorithm are employed, respectively, to decide on the location of data centers to minimize delay, energy consumption, costs and the emission of greenhouse gases. The solution proposed by Covas, Silva and Dias [15] also considers a multiple criteria decision that quantifies the social, economic, and environmental impact of candidate location for the data center. Their proposal employs the method ELECTRIC TRI to classify all criteria; the solution was validated with a local provider. These solutions for cloud data centers cannot be applied for the facility location for fog computing since cloud data centers are centralized, while fog nodes are distributed, the decision must thus consider other aspects.

The placement of cloudlets has been explored in previous papers [16,17]. Jia et al. [16] determined the location of cloudlets to reduce the delay of user tasks. Fan and Ansari [17] included the cloudlet cost in the decision. Using an optimization model, they showed that their solution can reduce deployment cost as long as additional delays are acceptable. Since these papers do not consider the existence of the cloud, they fail to consider various applications with different latency requirements.

Previous work on the facility location problem in contexts [18,19,20] other than clouds and cloudlets are reviewed here since they share some characteristics with the problem discussed in this paper. There have been few approaches to the facility location problem in relation to the time axis. One approach is that in multi-period [18]. In such a problem, facilities are created in different time slots. Clients can choose any facility, but, once they initiate service at a given facility, they cannot change this facility. This solution, however, does not cover the node location problem in fog, since it considers neither localized demands nor limited capacity facilities.

Oliveira and Viana [19] presented a solution for WiFi hotspot location that maximizes the offloaded traffic for the limited number of deployed hotspots. The solution employs a time varying graph which relates mobile users and points of interest in a metropolitan area. Based on this graph, points of interest are selected for the deployment of hotspots. Results show that a small number of hotspots are sufficient to provide adequate offloading. The proposal in the present paper considers time-varying traffic demands but also analyzes various classes of requests.

Planning a fog infrastructure requires knowledge about the demand in different regions in order to establish fog nodes. One example is provided by the cellular network data that Telecom Italia collected in the region of Milan and Trentino in Italy in 2013 [21]. This dataset contains user demands by network cell and aggregated in 10-minute intervals during a two-month period. Given the reduced window interval and the separation into several non-overlapping areas, this dataset was capable of capturing information on user demands and their mobility, presenting user demands as a function of time. Using this dataset, Chen et al. [20] studied the problem of clustering base stations to share Cloud Radio Access Network (C-RAN) resources. The solution aims at clustering neighbor base stations with complementary traffic patterns, so that the workload processed in the C-RAN is balanced, requiring fewer resources. Results show that this clustering scheme reduces deployment costs as well as energy consumption. The work proposed in this paper also employs this dataset [21].

This paper introduces a formulation for the problem of locating fog nodes taking into consideration human mobility [20,21] and the location problem [13,14,15,16,17,18,19]. Demands variable over time are employed to capture user mobility. Furthermore, different types of workload are taken into account, an issue which is relevant in the interaction between the fog and the cloud.

## 3. System Model

This section details the model for the system considered in this paper as well as the fog node location problem. The system is composed of a cloud and various fog nodes, hierarchically organized in three layers: cloud, fog, and end-user devices. The cloud can be accessed by any device. The fog layer is formed by fog nodes, with each fog node having a limited area of coverage. A fog node is a small facility which hosts dedicated servers capable of processing end-user workload. Compared to the cloud, fog node resources are limited. End-user devices are in the lowest layer. User devices can move along the lowest level. These devices run several types of applications with different latency requirements. A user can access either the closest fog node (as long as this fog node covers the user) or the cloud. The decision of where to process user workloads depends on the workload itself. In this paper, the workloads are classified into two classes: fog (strict latency) and cloud (flexible latency) workloads. The former represents workloads which can only be hosted in a nearby fog node due to the latency requirements, while the latter can be processed in either the fog or the cloud.

Supporting client applications (workload) requires making the decision about the location of the fog nodes. To make these decisions, the selection of potential locations for receiving dedicated servers is necessary. Then, the selection of fog node locations can be made on the basis of the history of demands in these locations.

Each fog node is characterized by its location and the number of servers. The greater is the number of servers, the larger is the capacity of the fog node. To increase the total workload processed, strict latency workload should be first assigned for execution on fog servers. The remaining capacity of the fog nodes can then be used to process flexible latency workload. Executing flexible latency workload in the fog can reduce the latency for this type of load, thus enhancing user experience. Moreover, reducing the demands on the cloud allows the turning off of servers in the data center to save energy [22].

**Figure 1:**
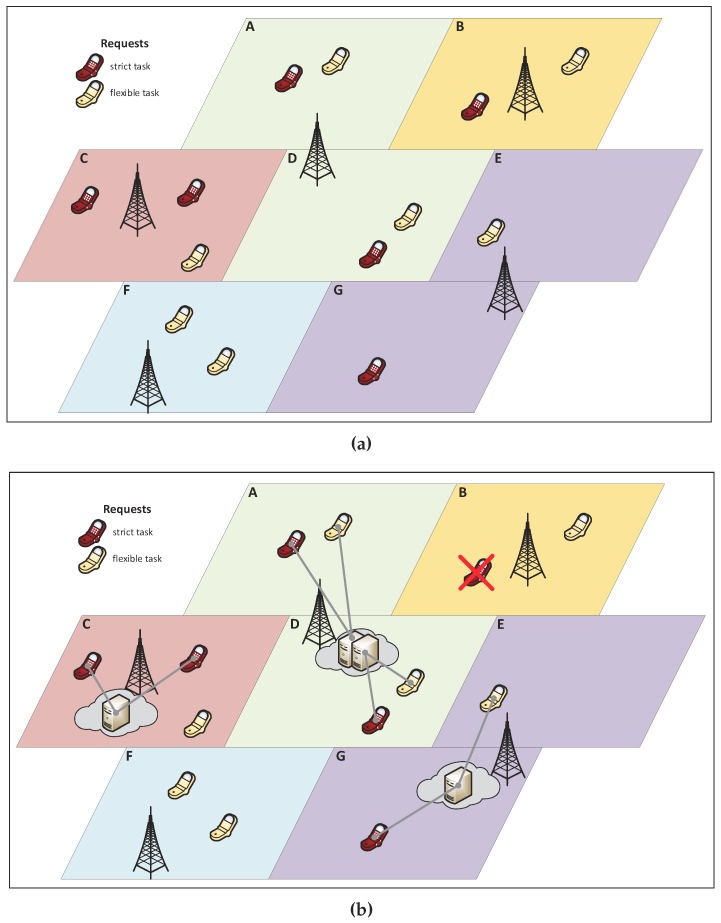
Example of fog location decision making: (**a**) possible locations and available number of servers; and (**b**) fog nodes decided and requests served by them.

The system considered in this paper assumes that both strict and flexible workloads vary over time. Without loss of generality, a discrete-time model has been adopted. Figure 1a illustrates the node location problem. This figure presents a segment of a city, divided into seven areas, identified by letters A–G, with end users served by five base stations (BS). Regions A and D are served by the same BS, another BS processes the requests made in Regions E and G, and the remaining regions are each served by an individual BS. A cellphone represents a request and the color associated with it identifies the type of request (strict or flexible). BSs are considered to be possible locations for hosting a fog node. Suppose that the provider can employ up to four servers, and each server can host two requests at the same time. One possible solution for this scenario is shown in Figure 1b. Three fog nodes have been created, one with two servers in the BS in Region D, and two nodes with a single server in Regions C and G. The fog node in D can serve both strict and flexible requests in its coverage area. The fog node in C serves strict requests in its area, as well as a flexible request, which could not be served using the cloud resources. In Region B, strict requests are blocked, since no fog node is available.

This example provides a snapshot of user positions. In the problem considered in this paper, end users can change their position dynamically, thus leading to different occupation of devices over time in each region. Consequently, the deployed infrastructure must be efficient for the service over time, not only during a specific time interval.

## 4. Fog Location Model

The solution proposed for the fog node location problem is given by a multicriteria mixed-integer linear programming formulation. The goal is to process most of the strict workload in the fog nodes using the minimum number of servers possible to reduce the overall cost. Moreover, the unused capacity of fog nodes should be used for the processing of flexible latency workload to further reduce the latency of users with this type of workload. In this section, the formulation of the optimization problem is presented first in Section 4.1, followed by the explanation of the selection of a Pareto-optimal solution in Section 4.2, and, finally, a numerical example is given to illustrate the proposed model in Section 4.3.

### 4.1. Mathematical Model

The notation used in the model is presented in Table 1. The provider budget constraint is given by *N*, the maximum number of dedicated servers to be employed in the fog nodes, each of them with capacity *R*. L and T are the location and time interval sets, respectively. $f_{lt}$ and $c_{lt}$ are also part of the input and represent, respectively, the strict latency and flexible latency workload demands at location *l* and time *t*. The solution consists of $\alpha_{l}$, the number of dedicated servers deployed at each location. Additionally, variables $f\;\;f_{lt}$, $c\;f_{lt}$, and $c\;c_{lt}$ indicate where each demand is processed (fog or cloud) for all locations and time periods.

The multi-objective formulation has three objective functions:

(1) $$\mathrm{maximize}\sum_{l\in L}\sum_{t\in T}\left(f\;\;f_{lt}\right)$$

(2) $$\mathrm{minimize}\sum_{l\in L}\alpha_{l}$$

(3) $$\mathrm{maximize}\sum_{l\in L}\sum_{t\in T}\left(c\;f_{lt}\right)$$

The constraints of the problem are the following:

(4) $$\sum_{l\in L}\alpha_{l}\leq N$$

(5) $$f\;\;f_{lt}+c\;f_{lt}\leq \alpha_{l}\cdot R,l\in L,t\in T$$

(6) $$f\;\;f_{lt}\leq f_{lt},l\in L,t\in T$$

(7) $$c\;f_{lt}+c\;c_{lt}=c_{lt},l\in L,t\in T$$

(8) $$c\;f_{lt}\geq 0,l\in L,t\in T$$

(9) $$f\;\;f_{lt}\geq 0,l\in L,t\in T$$

(10) $$\alpha_{l}\geq 0,l\in L$$

Equation (1) maximizes the processing of workload of the strict type on the fog nodes, i.e., it guarantees the maximum number of users for each time slot. To achieve this goal, the number of fog nodes at each location is determined using the minimum possible number of servers with Equation (2). Moreover, Equation (3) ensures that servers are deployed to locations where the remaining capacity can be used to boost the processing of flexible latency workload in the fog.

The constraints of the model are explained by the following. The constraint in Equation (4) limits the number of deployed servers to the total number of available devices *N*. The constraint in Equation (5) guarantees that the workload hosted in each fog node (sum of strict and flexible workload) is never greater than its capacity (number of servers multiplied by the capacity of a single server). The constraint in Equation (6) limits the strict latency workload processed at a fog node to the demand at that location. The constraint in Equation (7) guarantees that all flexible latency demand is met, whether at a local fog node or in the cloud. Finally, the constraints in Equations (8)–(10) set the minimum values for the decision variables.

### 4.2. Multicriteria Decision

The presented model is multicriterial, so that all possible solutions are the elements of a Pareto front. However, a single solution must be selected for the location problem. The multi-level programming approach was used to obtain the solution to the multicriteria formulation proposed in this paper. In this subsection, other approaches to solve multicriteria models are reviewed, and then the multi-level programming applied to the proposed formulation is explained.

There are various techniques for choosing a single solution from the Pareto front, such as scalarization, the ε-constraints method, goal programming, and multi-level programming [23]. Using scalarization, weights are assigned to each objective, and they are combined into a single objective. Such a solution is useful to evaluate trade-offs with different priorities for each objective. The ε-constraints method favors the main objective function, and the remaining objectives become constraints limited to given target values. Goal programming aims at finding values given by the user for each objective rather than optimizing them individually. Finally, under multi-level programming, the objectives are hierarchically ordered and sequentially optimized, so that neither an assignment of weights to the objectives nor changes in the constraints are necessary. The approach used by multi-level programming is explained as follows. The candidate solutions that optimize the first objective function are selected, giving, as a result, a subset of the Pareto front. Among the candidate solutions from this subset, the second objective is optimized, and so on, until all objectives have been evaluated. Employing multi-level programming is thus useful whenever objectives can be hierarchically organized.

In the fog node location problem, the service of end users is essential. Once this is achieved, the provider costs should be reduced and the usage of the remaining servers optimized. As a consequence of this order of priorities, the problem is appropriate to be solved using multi-level programming, which is the approach used in this paper, considering Equation (1) to be the main objective, followed by the objectives defined in Equations (2) and (3). Other methods can be employed for the solution, but they do not take into consideration the hierarchy between the objectives, either favoring a single objective or a trade-off, which do not make them adequate for the problem in this paper. However, to evaluate multiple solutions, degradation in some of the objectives is evaluated, as explained in Section 5.2.

### 4.3. Numerical Example

**Figure 2:**
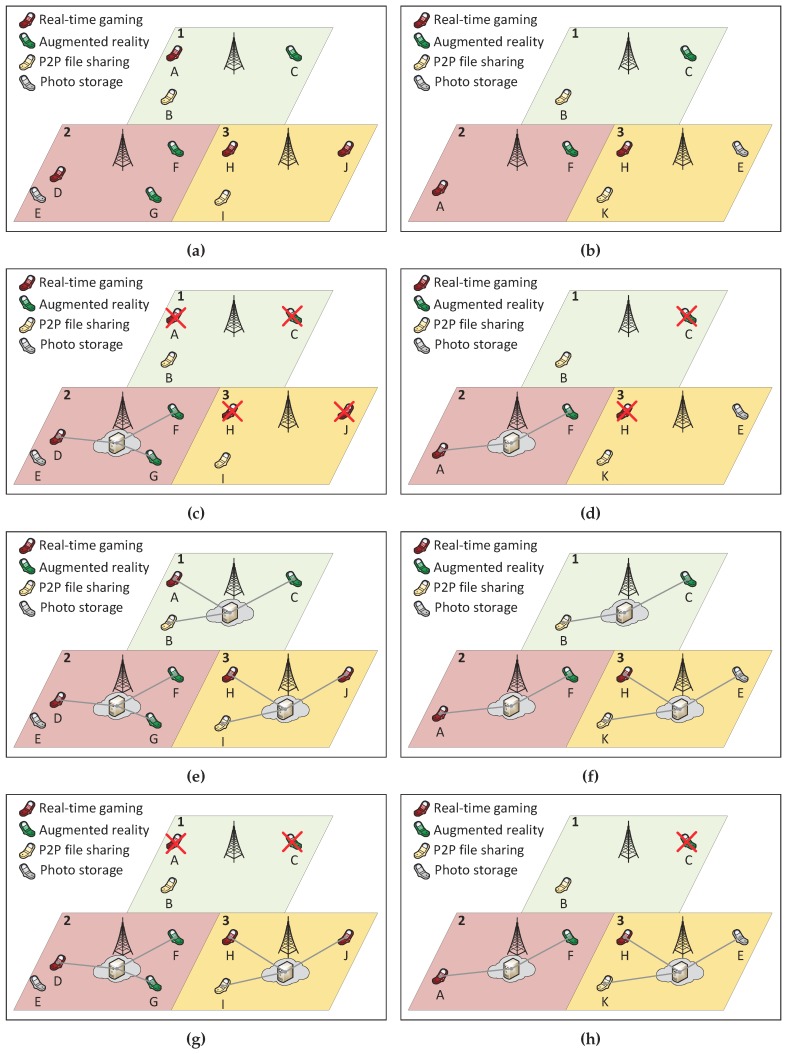
Numerical example of fog location decision making: (**a**) input at the first time slot; (**b**) input at the second time slot; (**c**) solution for N=1 at the first time slot; (**d**) solution for N=1 at the second time slot; (**e**) solution for N=4 at the first time slot; (**f**) solution for N=4 at the second time slot; (**g**) solution for N=2 at the first time slot; and (**h**) solution for N=2 at the second time slot.

To numerically illustrate the proposed MILP model, consider the example displayed in Figure 2a,b, which shows a snapshot of users’ position at time slot 1 and 2, respectively, for a small region of a city. There are three locations (1, 2 and 3) served by base stations; such BSs are candidates for the deployment of fog nodes. Eleven users, identified by letters A–K, execute four different applications in their smartphone. Users A, D, H, and J play a real-time game, while Users C, F, and G execute an augmented reality application, both applications require a fog node due to the low latency constraints. The remaining users execute applications which can be either processed in the fog or in the cloud due to their flexible latency requirements: Users B, I, and K share files in a P2P network while User E takes photos and then processes and stores them externally. Users that share files can take advantage of the fog by sharing files between them without the delay imposed by the cloud; in this case, the fog node coordinates the operations. For User E, the presence of a fog node allows the image processing in the fog, which reduces the transmission of large raw files to the cloud. Although the flexible latency applications can be boosted with a fog node, their processing can be realized by the cloud. Additionally, this example presents mobility: from time slot 1 to 2, User A goes from Location 1 to 2; User E from 2 to 3; Users D, G, and I leave the displayed area; and the new User K arrives in Location 3 at the second time slot.

The presented scenario can be mapped into the input of the fog node location problem. There are three base stations in Figure 2, thus L=3 and L={1,2,3}, and only two time slots are considered, so that T=2 and T={1,2}. Suppose that each fog server can host up to three requests at the same time (R=2). Considering the requests displayed in Figure 2a,b, the strict and flexible workloads assume the following values: $f_{11}=2$, $f_{21}=3$, $f_{31}=2$, $f_{12}=1$, $f_{22}=2$, $f_{32}=1$, $c_{11}=1$, $c_{21}=1$, $c_{31}=1$, $c_{12}=1$, $c_{22}=0$, and $c_{32}=2$. All these values are used as input to the problem. The values of *N* are varied to exemplify the priority of each objective in the multi-level programming approach.

The main goal of the formulation is to serve all strict workload (the objective function in Equation (1)). To illustrate that, consider N=1, i.e., only one fog node with a single server can be deployed. In this case, a fog node is created at Location 2 ($\alpha_{1}=\alpha_{3}=0$ and $\alpha_{2}=1$) since it produces 5 for Equation (1). If $\alpha_{1}=1$ or $\alpha_{3}=1$, the produced values (3 in both cases) would not be optimal. The solution for N=1 is displayed in Figure 2c,d.

The effect of the objective function in Equation (2) is noticed for N=4. In this case, all locations can be covered by fog nodes with a single server ($\alpha_{1}=\alpha_{2}=\alpha_{3}=1$), case in which no strict application is blocked and the value obtained for Equation (1) is 11. The addition of the fourth server in any fog node does not increase the value of Equation (1), thus the objective function in Equation (2) limits the employed servers to 3 to avoid extra costs with the infrastructure deployment. The scenario described in this paragraph is illustrated in Figure 2e,f.

Finally, a practical example of the effect of the objective function in Equation (3) happens for N=2 (Figure 2g,h). As discussed earlier, the most demanded fog node is the one in Location 2 ($\alpha_{2}=1$), thus, when there is an extra server available, the decision is which of the other locations should host a fog node, $\alpha_{1}=1$ or $\alpha_{3}=1$. Either option produces the same value for Equations (1) and (2): 8 and 2, respectively. Therefore, the objective function in Equation (3) is evaluated. If $\alpha_{1}=1$, then Equation (3) assumes the value 2, while $\alpha_{3}=1$ leads to the value 3. Thus, the fog node is deployed in Location 3, allowing Users E, I, and K to use the fog instead of the cloud, improving the latency of the delivered service.

## 5. Performance Evaluation

The mixed-integer linear programming model was coded using the Gurobi Optimizer solver. The time-varying demands used as input to the problem were obtained from two datasets [21,24], as explained in Section 5.1. Using the MILP model, solutions which provide alternative trade-offs were also evaluated and are described in Section 5.2. Numerical results are discussed in Section 5.3.

### 5.1. Workload

The values of variables $f_{lt}$ and $c_{lt}$ (fog/strict and cloud/flexible workload demands) were taken from the dataset in [21]. Every time a mobile user required services from a telecommunications provider, a Call Detail Record was recorded in the metropolitan area of Milan during a two-month period. The geographical area was divided into a 100 × 100 grid, in which each cell has information on the Short Message Service (SMS) messages received and sent, phone calls made and received, and Internet usage. These data were aggregated into 10-minute intervals. In this paper, the Internet usage information models the workload demands since it represents a variety of mobile applications, different from calls and SMS. This dataset was chosen since it provides real records of a city accounting for user mobility.

In the dataset [21], demands are separated into geographical cells, but users actually request services from a base station, which may not be in the cell area. Correspondingly, some base stations serve a larger number of cells (larger areas). The set of locations L is, therefore, the set of areas covered by the antennas, the location of which was determined by the OpenCellId project [24], an open database containing information about base stations worldwide collected by mobile users. This database has comprehensive data and has been employed in previous work reported in the literature [25]. The location of all base stations was obtained by filtering the existing base stations in the period of the Milan dataset [21]. The workload of each cell was mapped to the closest base station, as in [20]. In the case of multiple base stations inside a cell, the workload is equally balanced on these BSs. Thus, L is the set of base stations obtained from the OpenCellId project, and the workload of each cell from [21] is associated with the closest base station to define the values of $f_{lt}$ and $c_{lt}$. In this paper, a complete fog-cloud infrastructure is designed, so that all locations in L are considered, thus evaluating the complete metropolitan area of Milan. Although the solution can be evaluated on a smaller scale, results are presented for all locations.

The input to the problem consisted of *N*, *R*, L, T, $f_{lt}$, and $c_{lt}$. The capacity *R* of a server was fixed, and *N* was varied to evaluate solutions obtained under different budget constraints. The number of locations in L was determined using the OpenCellId dataset as explained above. Since the dataset [21] has data for two months, *T* was also varied to evaluate the solution under different lengths of planning intervals, from 1 h to 24 h. The proportion of fog and cloud requests was varied using three scenarios, namely P25, P50, and P75. In P25, 25% of the workload for an antenna was strict and 75% flexible. In the P50 scenario, the proportion was 50% for each type of request, and, in P75, the workload is 75% strict and 25% flexible. Table 2 summarizes the input values and the adopted scenarios.

### 5.2. Multi-Objective Solutions Allowing Degradation

The MILP model presented in Section 4 was coded using the multi-level programming approach; the solution was identified by OPT. Employing only OPT leads to a single solution for the problem. However, a fog provider can accept decreasing performance for one of the objectives if significant improvements are obtained for the other objectives, i.e., if an advantageous trade-off, for the multiple objectives, is achieved. Various solutions were evaluated that allowed degradation in some of the objective functions.

These solutions differ from OPT since they allow degradation of either the objective function in Equation (1) or the objective function in Equation (2). Solutions that allow degradation of the objective function in Equation (1) are identified by STRX, where *X* is the percentage value that can be degraded from the total served strict workload. By allowing degradation of the objective function in Equation (1), these solutions can employ fewer servers, thus reducing deployment costs. Degradation of the objective function in Equation (2) was also evaluated. SERX identifies the solutions that degrade the number of employed servers, i.e., they allow an increase in the number of servers in *X* % in relation to OPT to increase the amount of flexible workload processed in the fog. Since strict workloads are blocked if not served in the fog, applying STRX has a great impact on end users, thus only up to 20% of degradation was evaluated. Employing more servers, differently, does not prevent the execution of strict workloads, thus, up to 30% of degradation was evaluated for SERX. Table 3 summarizes all the solutions evaluated.

### 5.3. Numerical Results

In this subsection, the performance of the proposed solution is assessed. This evaluation showed how the solution improves fog service, reducing costs and dealing with the two types of workload. Furthermore, several scenarios with different traffic patterns and budget constraints were used to evaluate the efficiency of the solution under various conditions. First, the results produced by OPT using different planning intervals are discussed. Then, the results obtained under degradation are presented. Finally, different scenarios of traffic patterns (P25, P50, and P75) were evaluated. Three metrics were considered: acceptance ratio of strict latency workload, acceptance ratio of flexible latency workload in the fog, and the number of deployed servers. A 95% confidence interval is used in the graphs. In this section, STRX is used to refer to all solutions that allow degradation of the objective function in Equation (1) and SERX to all solutions that allow it for the objective function in Equation (2). Graphs of the strict latency acceptance ratio are in a logarithmic scale.

**Figure 3:**
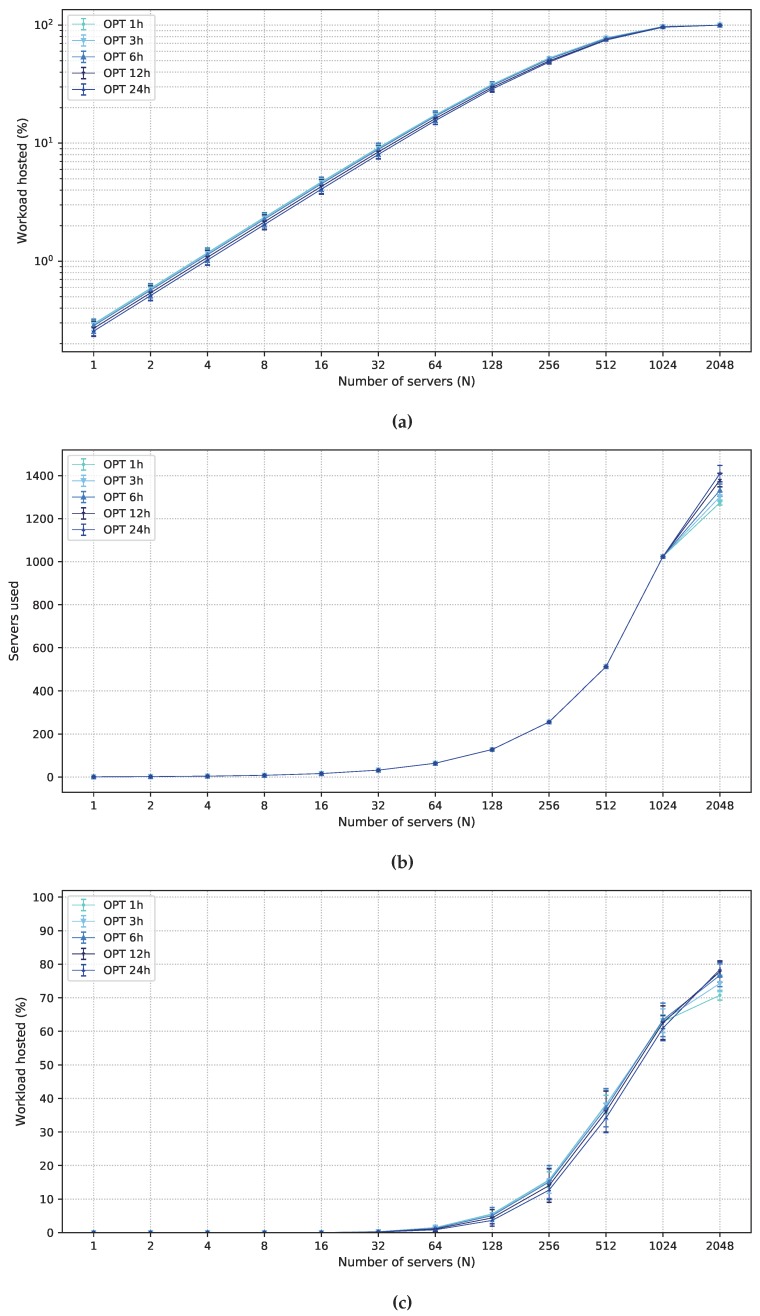
Results obtained for OPT under P50 scenario: (**a**) strict latency workload acceptance ratio; (**b**) average number of servers employed; and (**c**) flexible latency workload acceptance ratio in the fog.

OPT results are discussed for the P50 scenario in Figure 3. The larger is the number of available servers, the larger is the number of servers utilized (Figure 3b). This is a result of the main goal of the solution to serve the maximum number of strict workloads possible, which leads to more servers being used in the solution. For 1≤N≤1024, the available servers cannot cope with the entire strict latency workload since most of the available servers (*N* servers) are used. This causes the overlap of the curves for all planning intervals. For N≥2048, the available capacity is greater than the total demand, so the entire demand is met (Figure 3a), requiring between 1480 and 1710 servers. The number of required servers varies according to the planning interval: short planning intervals may not contain periods during which a location is crowded. Consequently, for longer intervals, a large number of periods of peak demand is present for several locations, which requires the deployment of a larger number of servers. Results for N>2048 are the same as those for N=2048 since the multi-level programming approach optimizes the entire served demand in Equation (1); hence a larger number of servers does not lead to any improvement in the strict latency workload service.

The ratio between flexible requests served in the fog and the total flexible workload is shown in Figure 3c. The extra capacity of fog nodes can be used to host the flexible workload, thus, when nearby 80% of the strict workload is served (N=512), more than 30% of the flexible workload can be executed in the fog, which improves the latency of end users as well as allows more flexibility in the energy management of the cloud data center. OPT maximizes flexible requests utilization of fog nodes (the objective function in Equation (3)) only after satisfying the objective functions in Equations (1) and (2). As a result, no new fog servers will be deployed to host only flexible workloads. Thus, for N=2048, between 60% and 80% of the flexible workload is hosted in the fog and the remainder in the cloud. If the order of objective functions in Equations (2) and (3) were reversed in the multi-level optimization, flexible workload allocation would be prioritized in the fog, but at a higher server deployment cost than that was in the original order.

The order of the curves changes in the interval 1024≤N≤2048 in Figure 3c due to the availability of a larger number of servers for N=2048 and longer planning intervals. For N≤1024, the available resources do not meet the full demands of the strict workload. Conversely, when N=2048, all strict workloads are processed, and powerful fog nodes tailored to the peak demands are produced. Consequently, during periods when strict demands are low, fog servers are used to host the flexible workload. Thus, solutions for larger planning intervals are capable of hosting more flexible workloads, explaining the difference in the order of the curves in Figure 3c for *N* between 1024 and 2048.

**Figure 4:**
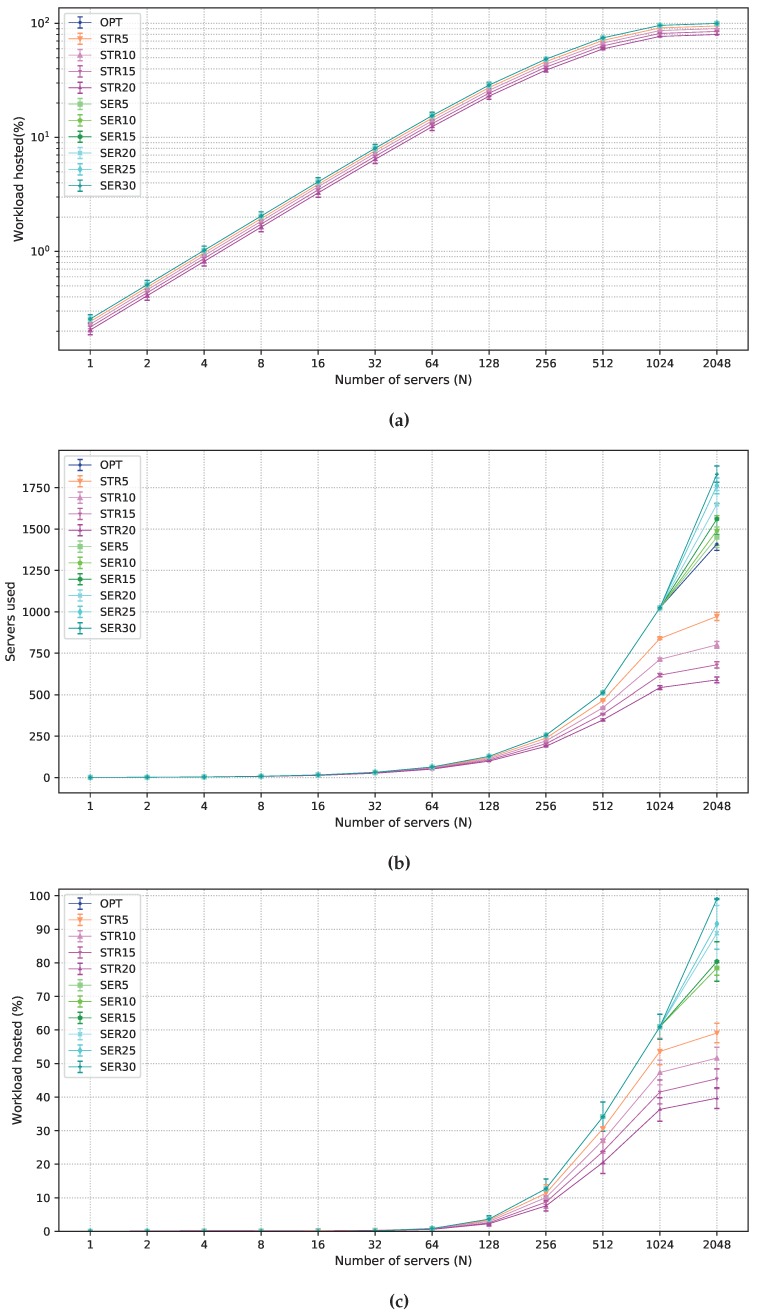
Results obtained for all solutions under P50 scenario. (**a**) strict latency workload acceptance ratio; (**b**) average number of servers employed; and (**c**) flexible latency workload acceptance ratio in the fog.

In the remainder of this section, results for 24 h planning intervals are shown. Using a larger interval results in more variation in demands in the considered locations. Hence, larger intervals are useful in planning long-term infrastructures. A comparison of OPT and the solutions which allow degradation in one of the objective functions is presented in Figure 4 for the P50 scenario. The acceptance ratio of strict latency workloads is shown in Figure 4a. The curves for OPT and all solutions that allow degradation in the objective function in Equation (2) overlap since it is optimized after the objective function in Equation (1). Curves corresponding to STRX are parallel to OPT in the log scale according to the allowed degradation, from 5% to 20%.

**Figure 5:**
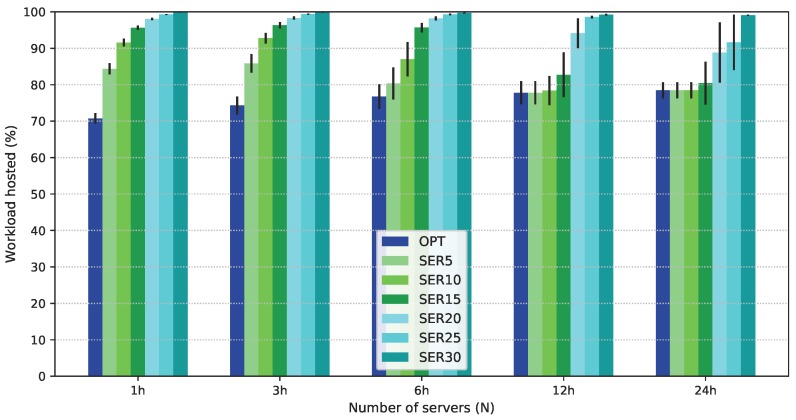
Flexible latency workload acceptance ratio in the fog for various planning intervals, N=2048 and P50.

Figure 4b shows the number of employed servers as a function of *N*. SERX deploys a larger number of servers than OPT and STRX. Since all servers are used for 1≤N≤1024, differences in the values obtained by OPT and SERX appear only for N=2048, when there is more capacity than that required for the strict workloads. For SERX, extra servers are employed to host more flexible latency workloads in the fog, as shown in Figure 4c. Notice, however, that an increase in the number of servers less than 15% results in a minimal increase in the flexible latency service in the fog. This is due to the distribution of demand across different locations. To explain this trend better, Figure 5 presents results for flexible latency workload in the fog for all values of the planning intervals considered (1 h, 3 h, 6 h, 12 h, and 24 h) and N=2048. For 1 h planning, an increase in the number of servers increases the acceptance ratio of flexible workloads in the fog. However, results for longer intervals show that small gains are obtained for degradation smaller than 15%. For small intervals, users are less mobile, which makes demands more uniform over all locations. Longer intervals, however, present peak demand periods on a larger number of locations. Thus, given that servers cannot be moved from one fog node to another, serving the total flexible demand in the fog requires a larger number of servers in many fog nodes, making the employment of SERX effective only when high degradation is allowed.

One important effect of STR5 is noticed for N=2048, where it reduces more than 400 servers in the solution in relation to OPT, which accounts to about 30% of savings in server costs (Figure 4b). This is due to the fact that the removal of one or two servers from each fog node does not lead to great blocking. Serving strict workloads is the main goal of optimization, hence most servers process mainly this type of workload. However, to fully process the demand, a fog node may have servers that remain idle or process only a small number of strict workloads. For example, during an interval facing peak demand, a fog node may need five servers to process all the strict demand, while most of the time only three or four servers would be sufficient. Thus, even if degradation in the objective function in Equation (1) is small, high infrastructure costs can be avoided if the fog node capacity is not tailored to the peak demands in the fog area. If the blocking of a small number of requests is acceptable, STRX becomes a viable solution.

**Figure 6:**
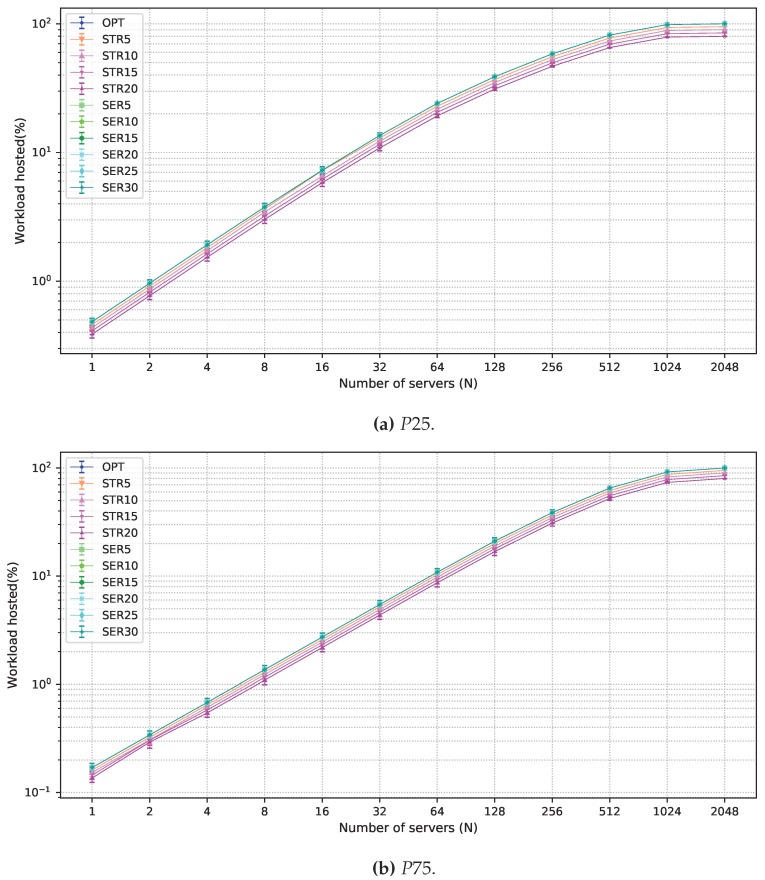
Results for strict latency workload acceptance under P25 and P75 scenarios.

Results for P25 and P75 scenarios are presented, yet for OPT, STRX, and SERX solutions and 24 h planning intervals. These results for the acceptance of strict latency workload are displayed in Figure 6. Results follow the same pattern of those under the P50 scenario. All solutions result in greater acceptance of strict latency workload under P25 than for those of P50, and less than those of P75. The former is explained by the reduction of the strict workload, making the available servers sufficient for dealing with a larger part of the strict demand. The opposite situation happens when there are more strict workloads, when the strict demands are harder to serve.

**Figure 7:**
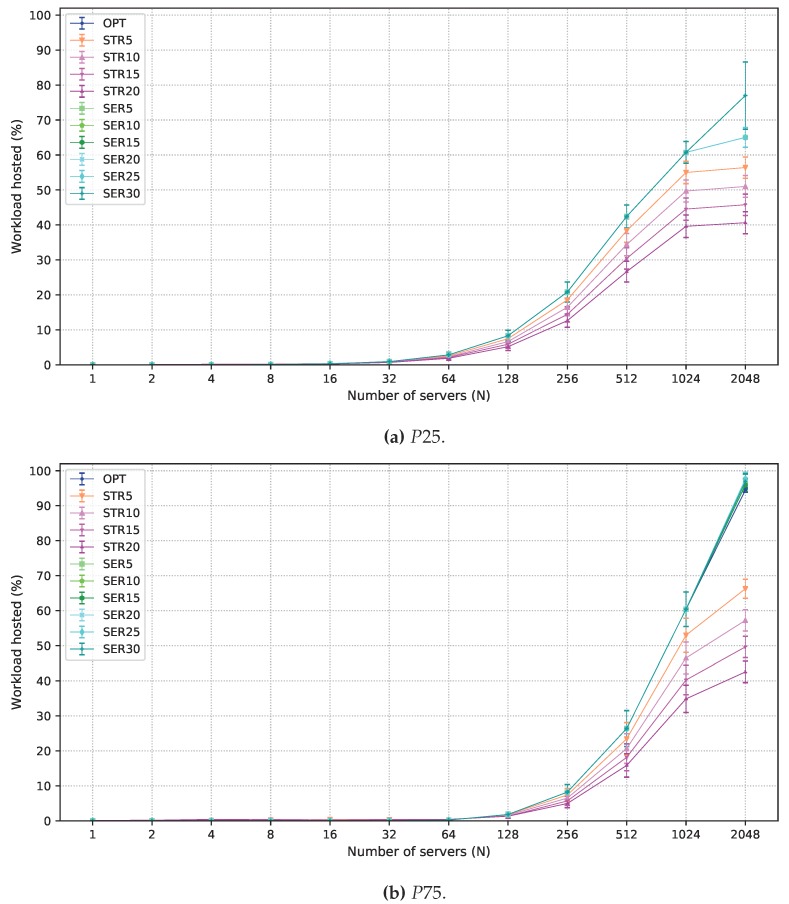
Results for flexible latency workload acceptance ratio under P25 and P75 scenarios.

**Figure 8:**
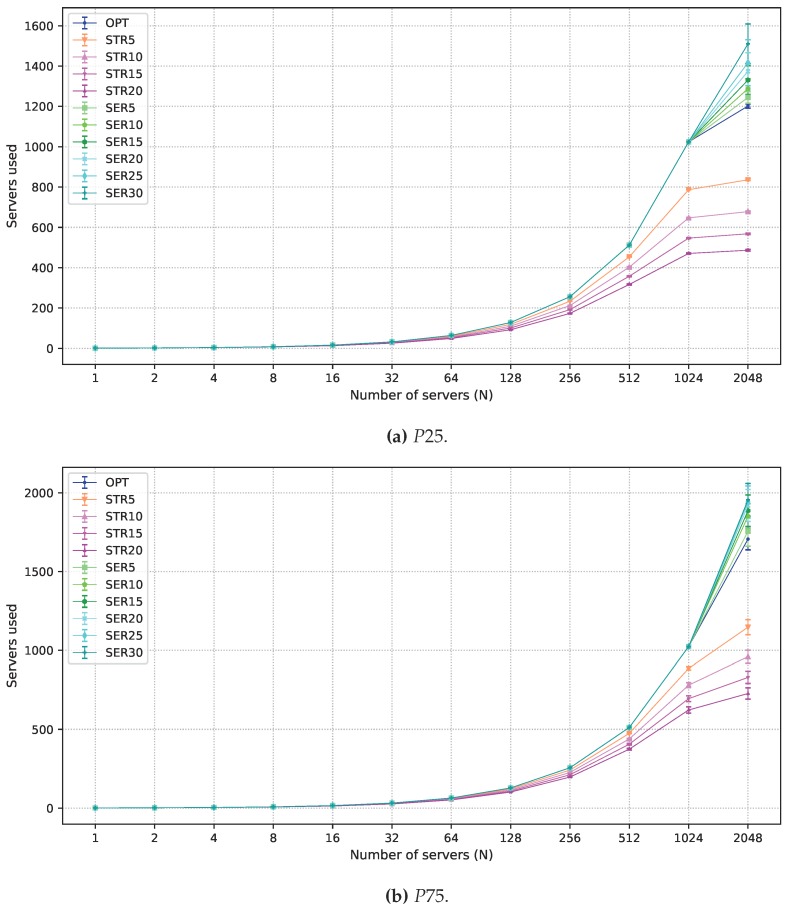
Results for the average number of servers employed under P25 and P75 scenarios.

The acceptance of flexible latency workloads in the fog and the number of employed servers are shown in Figure 7 and Figure 8, respectively, for both P25 and P75 scenarios. In the P25, there are less strict workloads. Accordingly, the total number of employed servers is reduced (Figure 8a), which also reduces the capacity available for hosting flexible workloads (Figure 7a). For P75, there is much more strict workload, which requires about 1700 servers (Figure 8b). The reduced demand for flexible latency workloads (Figure 7b) allows almost 100% processing of this demand in the fog nodes for OPT, and the employment of SERX under these circumstances leads to few gains. Finally, applying STR5 instead of OPT leads to savings about 30% for both the P25 and P75, as shown for P50. When the strict workload demand is high (P75), the absolute number of servers is higher, thus STR5 can reduce costs considerably with the infrastructure.

All results in this section were obtained using the Gurobi Optimizer solver. The execution time depends on the input size, mainly affected by *N* and the planning interval length. Scenarios with the largest inputs, high *N* and 24 h intervals, took less than 350 s, which is less than 1% of the planning interval length. Therefore, the proposed solution is feasible and, in the case of changes of demands, the location of fog nodes can be quickly recalculated.

This section has presented an evaluation of the results produced by the multicriteria optimization formulation employing multi-level programming proposed. Solutions considered hierarchical objectives with and without the allowance of degradation in one of the objective functions to optimize the others. The deployment of a fog infrastructure requires an analysis of all locations. Moreover, mobility of end users causes different regions to have demand peaks at different times, thus, in addition to the locations, the variation of demands must also be considered in the choice of the location. Given the priority of the multiple objectives, OPT represents the ideal solution. However, results for the other solutions produce interesting results: if the provider accepts the blockage of some users, the employment of STR5 leads to large savings with physical servers in the infrastructure, which leads to a potentially useful trade-off between service and deployment costs for the provider. The employment of SERX, on the other hand, is seldom useful since the number of servers in each fog node is fixed, although the demands are variable and distributed. All results can be obtained in a reasonable time using the proposed formulation.

## 6. Conclusions

This paper has studied the problem of locating fog node facilities in a fog-cloud scenario. The purpose is to decide on the locations where fog nodes should be deployed and the computing capacity of each node. This decision should improve the services delivered to end users, guaranteeing that most users who depend on the fog are served, and improving the network deployed to mitigate provider costs.

The problem was solved using a multicriteria MILP model. Two types of workload were defined to simulate different applications in a fog-cloud system with the evaluation using real data of user mobility. A multi-level programming approach was employed to obtain the final solution, in which the objectives were sequentially optimized.

The proposed MILP model was also evaluated when degradation is allowed of some of the objective functions. The results show that, due to the distribution of demands in relation to time and locations, infrastructure costs can be reduced if the provider is willing to accept the blockage of a limited number of users: allowing a 5% degradation in the strict latency workload service leads to about 30% savings in the number of servers for the infrastructure deployed. Furthermore, a substantial number of servers is needed to increase the processing of flexible demands in the fog, which significantly raises the deployment costs: allowing an increase of less than 15% in the number of servers has little effect on the service of flexible workload demands in the fog. Results obtained with the proposed MILP model can be quickly obtained, thus the solution can be recalculated when there are changes in the network.

One downside of the solution proposed in this paper is that it requires the previous workload demands for all candidate locations, which cannot be assumed for all providers, especially if a brand-new infrastructure is designed. Furthermore, the evaluation did not consider the deployment of a network to serve, for instance, a whole country, thus the evaluation of other datasets and bigger regional areas (states, provinces or countries) is a possibility for future work. Despite that, the solution presented in this paper can be applied to a metropolitan area inhabited by millions of citizens.

The distribution of demands in each location over time is a challenge for the fog provider. A solution to this problem in the context of C-RAN was to share resources of locations with complementary traffic patterns [20]. Therefore, solutions in the context of fog computing able to cope with this problem are recommended as future work. Another direction to extend this work is the consideration of other criteria, such as the energy consumption in the infrastructure or that spent by end users.

## Figures and Tables

**Table 1:** Notation used in the Fog Location Problem formulation.

**Input Parameters**	
**Notation**	**Description**
*N*	Maximum number of servers to be deployed
*R*	Capacity of a single server
*L*	Number of locations where a fog node can be created, $L\in N^{+}$
L	Set of all locations where a fog node can be created: L={1,2,⋯,L}
*T*	Total number of discrete time intervals, $T\in N^{+}$
T	Set of all discrete time intervals: T={1,2,⋯,T}
$f_{lt}$	Strict workload at location l∈L at time t∈T
$c_{lt}$	Flexible workload at location l∈L at time t∈T
**Decision variables**	
**Notation**	**Description**
$\alpha_{l}$	The number of servers created at location l∈L. If $\alpha_{l}=0$, no fog node is created at location *l*
$f\;\;f_{lt}$	Strict workload originating at location l∈L at time t∈T and hosted by the local fog node
$c\;f_{lt}$	Flexible workload originating at location l∈L at time t∈T and hosted by the local fog node
$c\;c_{lt}$	Flexible workload originating at location l∈L at time t∈T and hosted by the cloud

**Table 2:** Adopted values of input and scenarios.

Parameter	Values
*N*	1, 2, 4, 8, 16, 32, 64, 128, 256, 512, 1024, 2048
*R*	1000
L	L={1,2,...,L}, L=1150
T	T={1,2,...,T}, each t∈T represents a ten minute interval.
	*T* varies to represent 1 h, 3 h, 6 h, 12 h, and 24 h intervals
$f_{lt}$ and $c_{lt}$, l∈L, t∈T	Aggregated workload of cells for each base station
Proportion between strict and flexible workloads	P25: 25% of strict and 75% of flexible latency workload
	P50: 50% of strict and 50% of flexible latency workload
	P75: 75% of strict and 25% of flexible latency workload

**Table 3:** Solutions evaluated in this paper as well as objective function affected and level of degradation allowed.

	Objective Degraded	Level of Degradation
OPT	—	—
STR5	Equation (1)	5%
STR10	Equation (1)	10%
STR15	Equation (1)	15%
STR20	Equation (1)	20%
SER5	Equation (2)	5%
SER10	Equation (2)	10%
SER15	Equation (2)	15%
SER20	Equation (2)	20%
SER25	Equation (2)	25%
SER30	Equation (2)	30%
